# Supplementary figures and images for: Network pharmacology and molecular-docking-based strategy to explore the potential mechanism of salidroside-inhibited oxidative stress in retinal ganglion cell
Source: PLoS One. 2024 Jul 5;19(7):e0305343. doi: 10.1371/journal.pone.0305343 (PMC11226129; doi:10.1371/journal.pone.0305343)

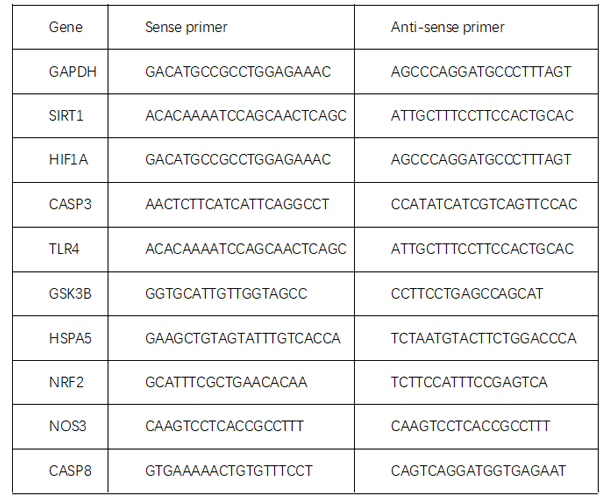

Supplement: S1 Fig — (TIF) [file pone.0305343.s001.tif]

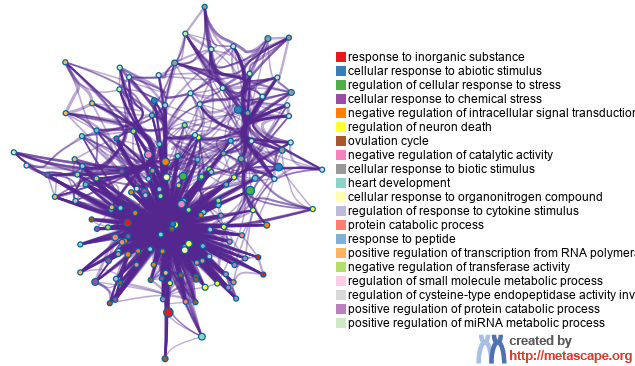

Supplement: S1 File — All raw data required to replicate the results of study were listed in this file. (ZIP) [file pone.0305343.s002.zip › original data/BP/Enrichment_GO/ColorByCluster.png]

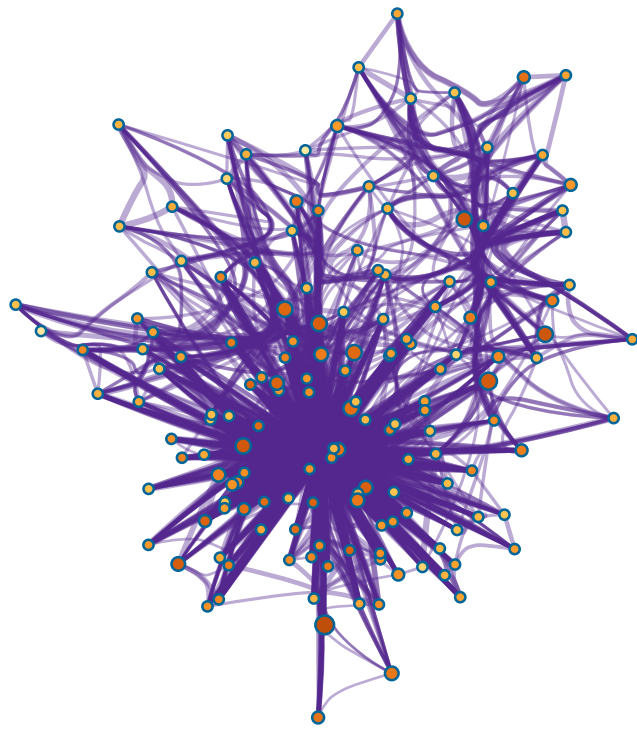

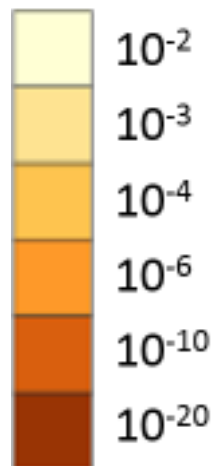

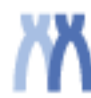 created by  
<http://metascape.org>

Supplement: S1 File — All raw data required to replicate the results of study were listed in this file. (ZIP) [file pone.0305343.s002.zip › original data/BP/Enrichment_GO/ColorByPValue.pdf]

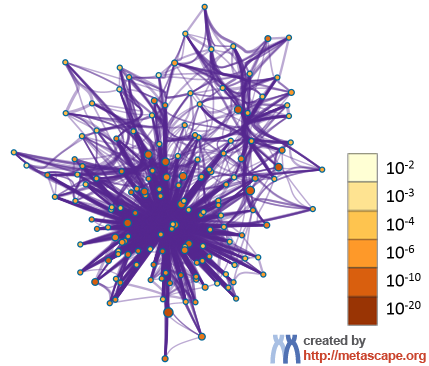

Supplement: S1 File — All raw data required to replicate the results of study were listed in this file. (ZIP) [file pone.0305343.s002.zip › original data/BP/Enrichment_GO/ColorByPValue.png]

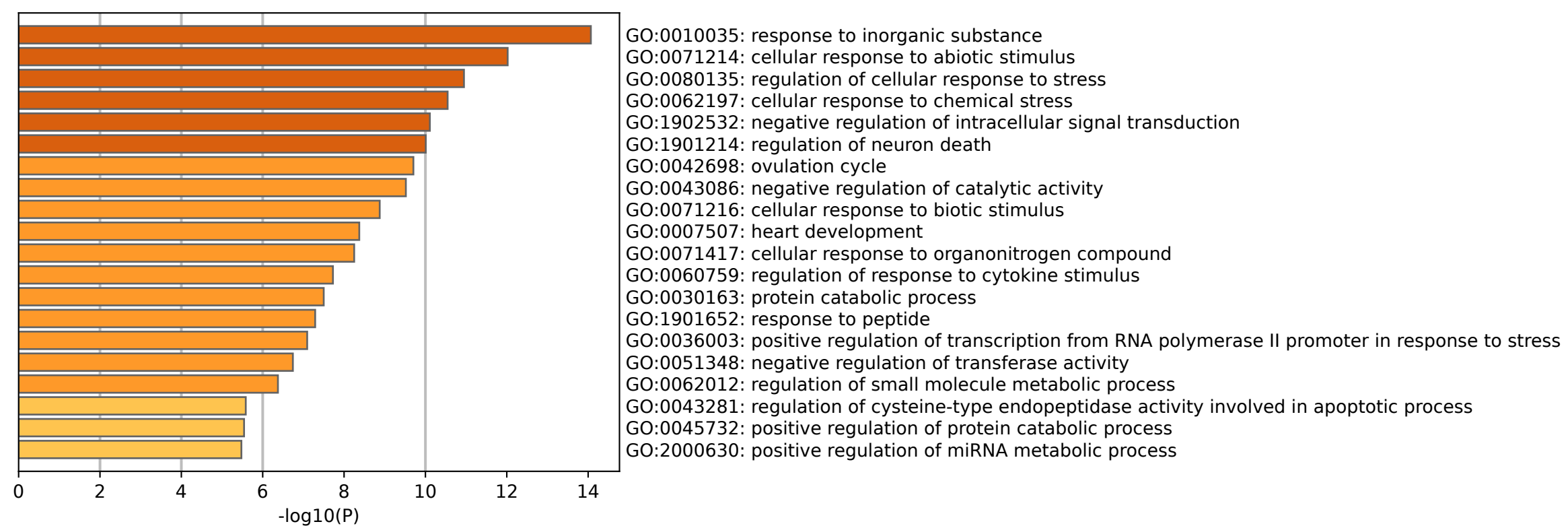

Supplement: S1 File — All raw data required to replicate the results of study were listed in this file. (ZIP) [file pone.0305343.s002.zip › original data/BP/Enrichment_heatmap/HeatmapSelectedGO.pdf]

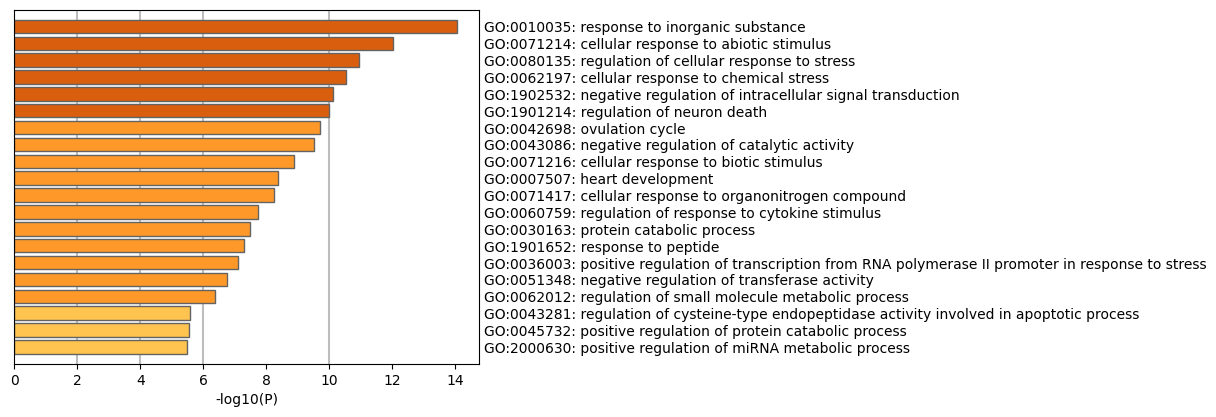

Supplement: S1 File — All raw data required to replicate the results of study were listed in this file. (ZIP) [file pone.0305343.s002.zip › original data/BP/Enrichment_heatmap/HeatmapSelectedGO.png]

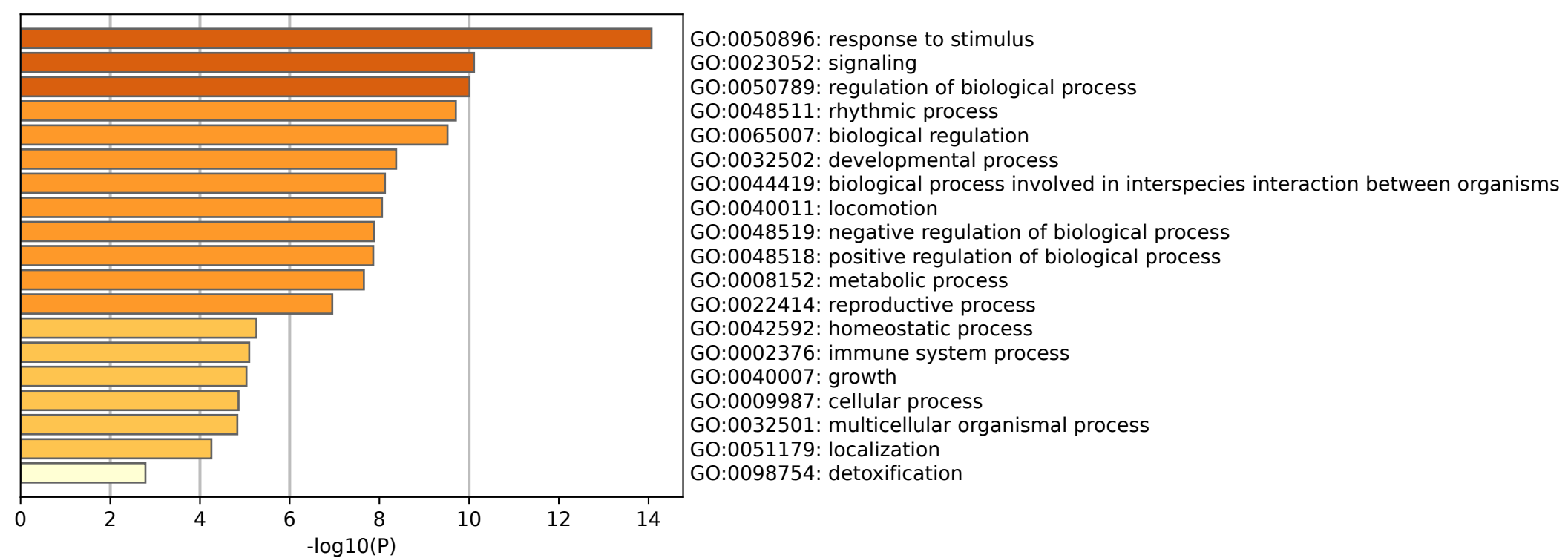

Supplement: S1 File — All raw data required to replicate the results of study were listed in this file. (ZIP) [file pone.0305343.s002.zip › original data/BP/Enrichment_heatmap/HeatmapSelectedGOParent.pdf]

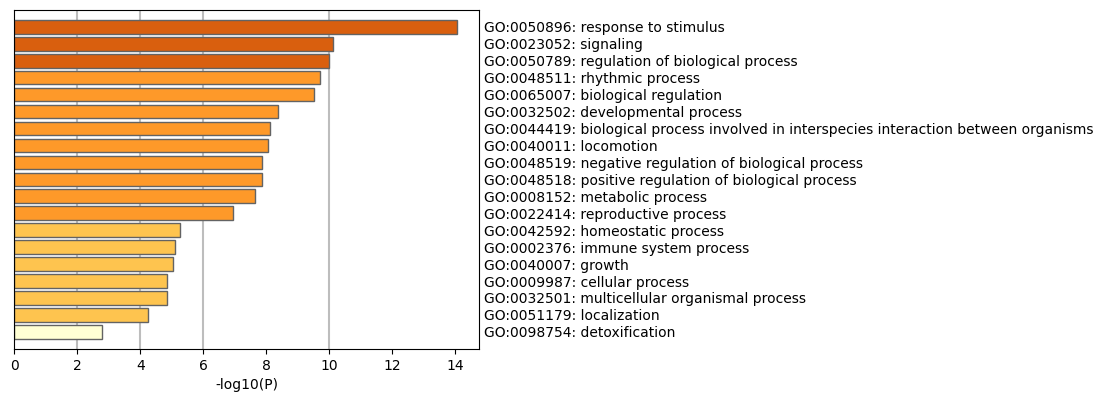

Supplement: S1 File — All raw data required to replicate the results of study were listed in this file. (ZIP) [file pone.0305343.s002.zip › original data/BP/Enrichment_heatmap/HeatmapSelectedGOParent.png]

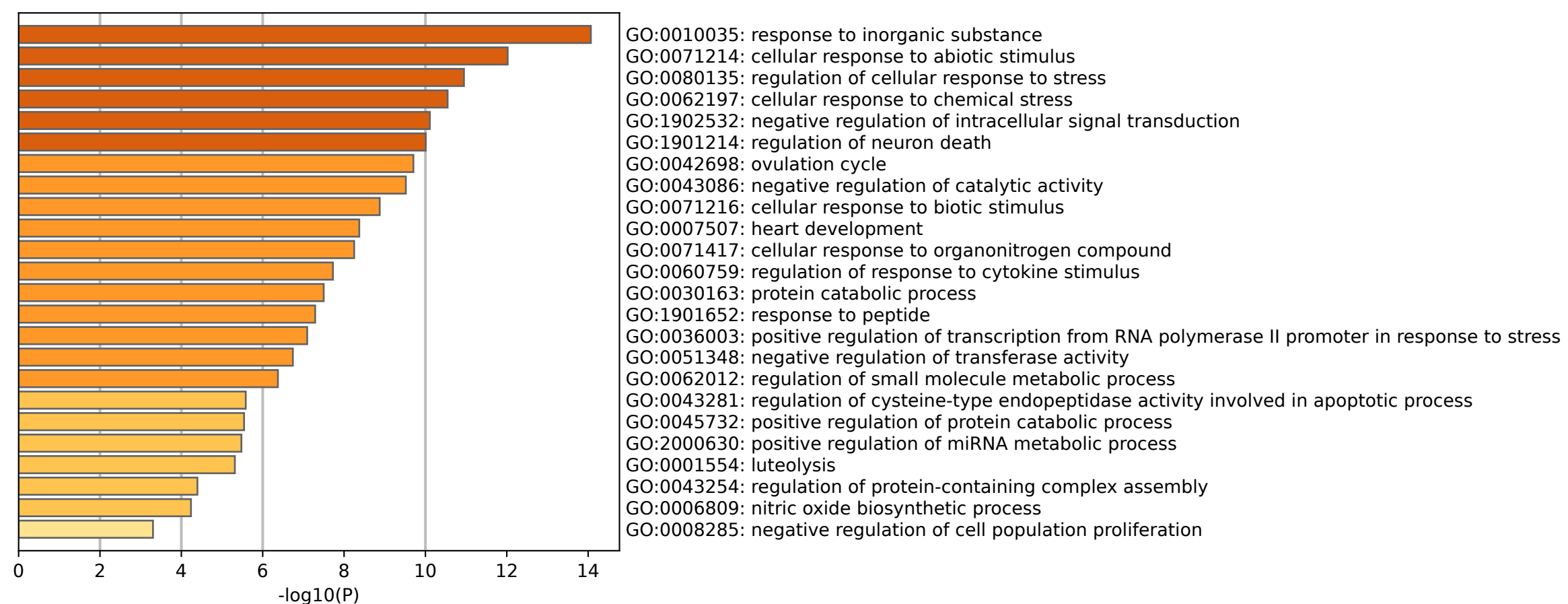

Supplement: S1 File — All raw data required to replicate the results of study were listed in this file. (ZIP) [file pone.0305343.s002.zip › original data/BP/Enrichment_heatmap/HeatmapSelectedGOTop100.pdf]

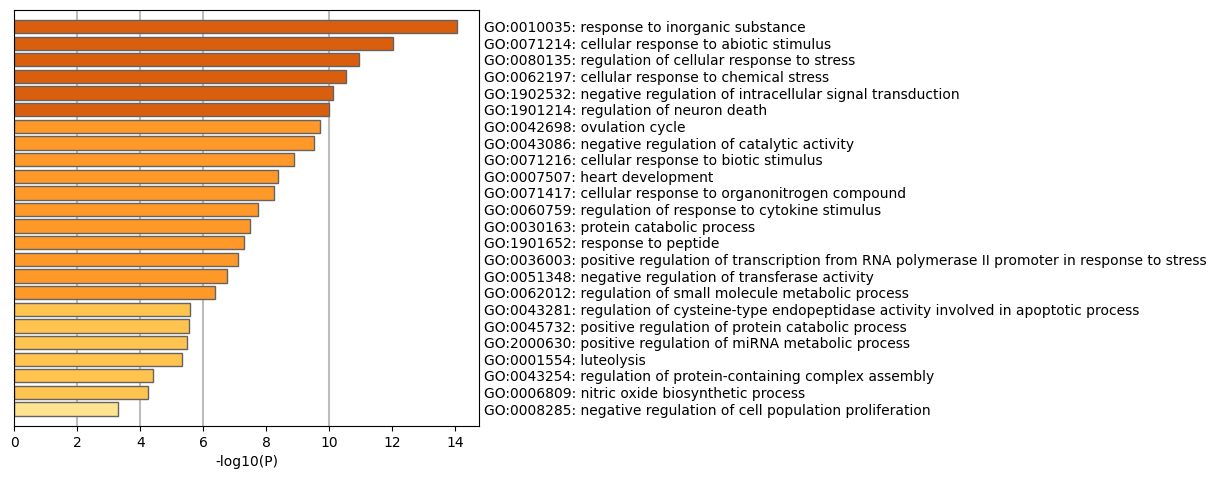

Supplement: S1 File — All raw data required to replicate the results of study were listed in this file. (ZIP) [file pone.0305343.s002.zip › original data/BP/Enrichment_heatmap/HeatmapSelectedGOTop100.png]

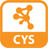

Supplement: S1 File — All raw data required to replicate the results of study were listed in this file. (ZIP) [file pone.0305343.s002.zip › original data/BP/icon/CYS48.png]

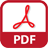

Supplement: S1 File — All raw data required to replicate the results of study were listed in this file. (ZIP) [file pone.0305343.s002.zip › original data/BP/icon/PDF48.png]

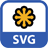

Supplement: S1 File — All raw data required to replicate the results of study were listed in this file. (ZIP) [file pone.0305343.s002.zip › original data/BP/icon/SVG48.png]

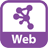

Supplement: S1 File — All raw data required to replicate the results of study were listed in this file. (ZIP) [file pone.0305343.s002.zip › original data/BP/icon/WEB_CYS48.png]

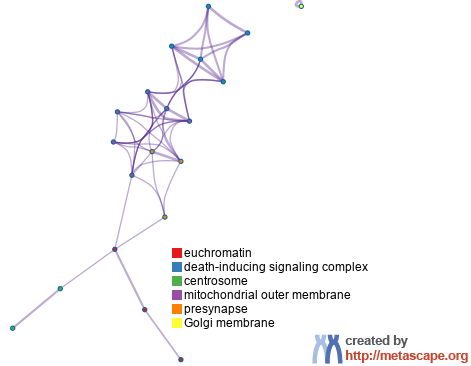

Supplement: S1 File — All raw data required to replicate the results of study were listed in this file. (ZIP) [file pone.0305343.s002.zip › original data/CC/Enrichment_GO/ColorByCluster.png]

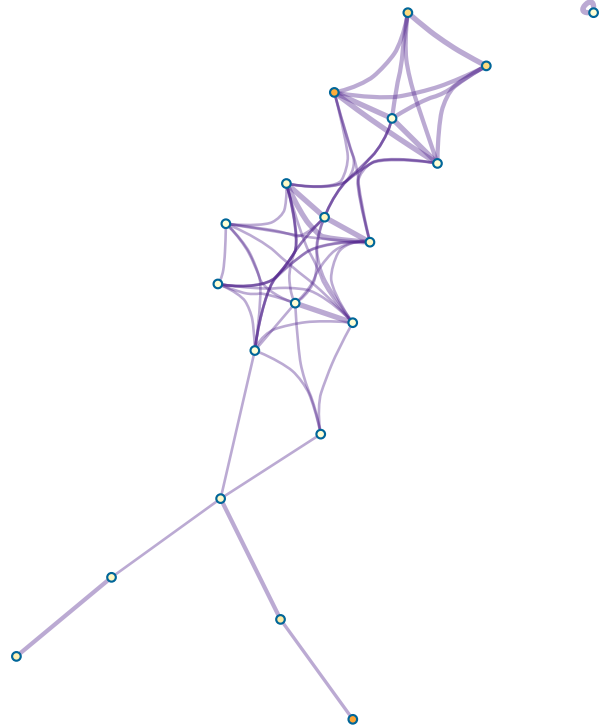

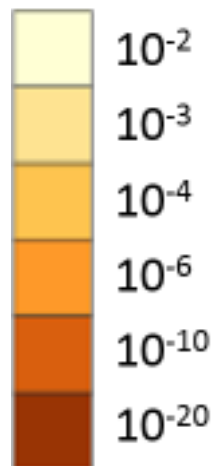

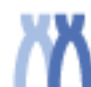 created by  
<http://metascape.org>

Supplement: S1 File — All raw data required to replicate the results of study were listed in this file. (ZIP) [file pone.0305343.s002.zip › original data/CC/Enrichment_GO/ColorByPValue.pdf]

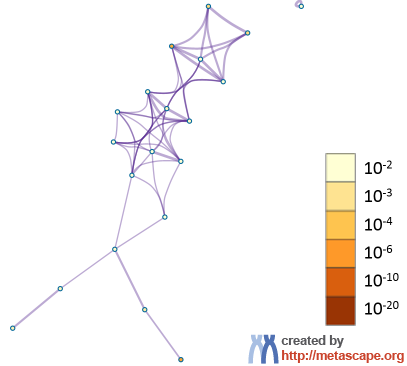

Supplement: S1 File — All raw data required to replicate the results of study were listed in this file. (ZIP) [file pone.0305343.s002.zip › original data/CC/Enrichment_GO/ColorByPValue.png]

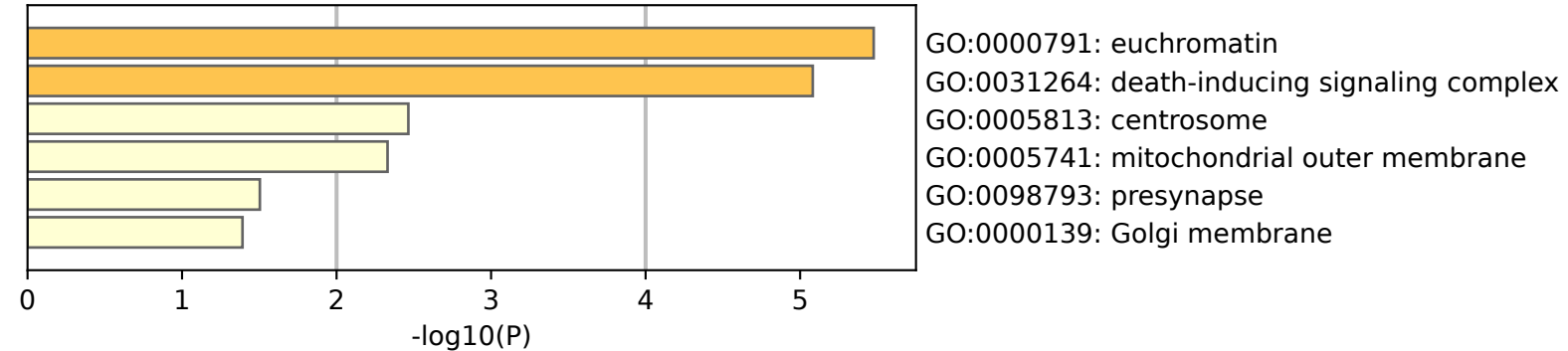

Supplement: S1 File — All raw data required to replicate the results of study were listed in this file. (ZIP) [file pone.0305343.s002.zip › original data/CC/Enrichment_heatmap/HeatmapSelectedGO.pdf]

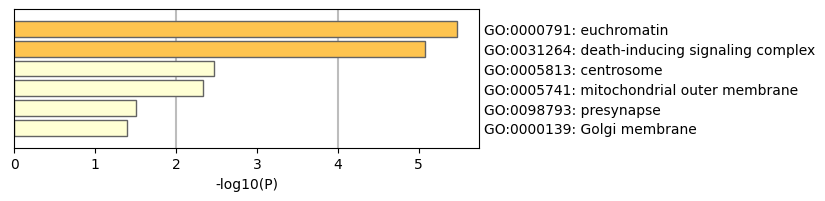

Supplement: S1 File — All raw data required to replicate the results of study were listed in this file. (ZIP) [file pone.0305343.s002.zip › original data/CC/Enrichment_heatmap/HeatmapSelectedGO.png]

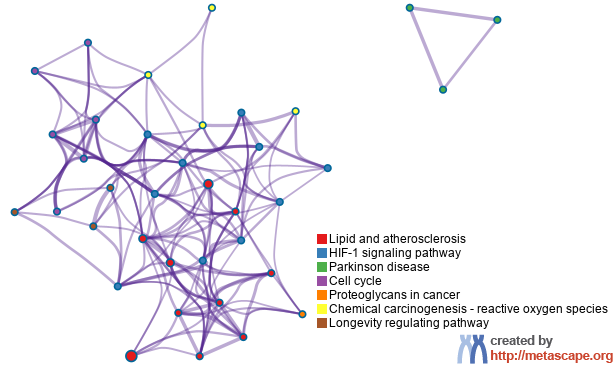

Supplement: S1 File — All raw data required to replicate the results of study were listed in this file. (ZIP) [file pone.0305343.s002.zip › original data/KEGG/Enrichment_GO/ColorByCluster.png]

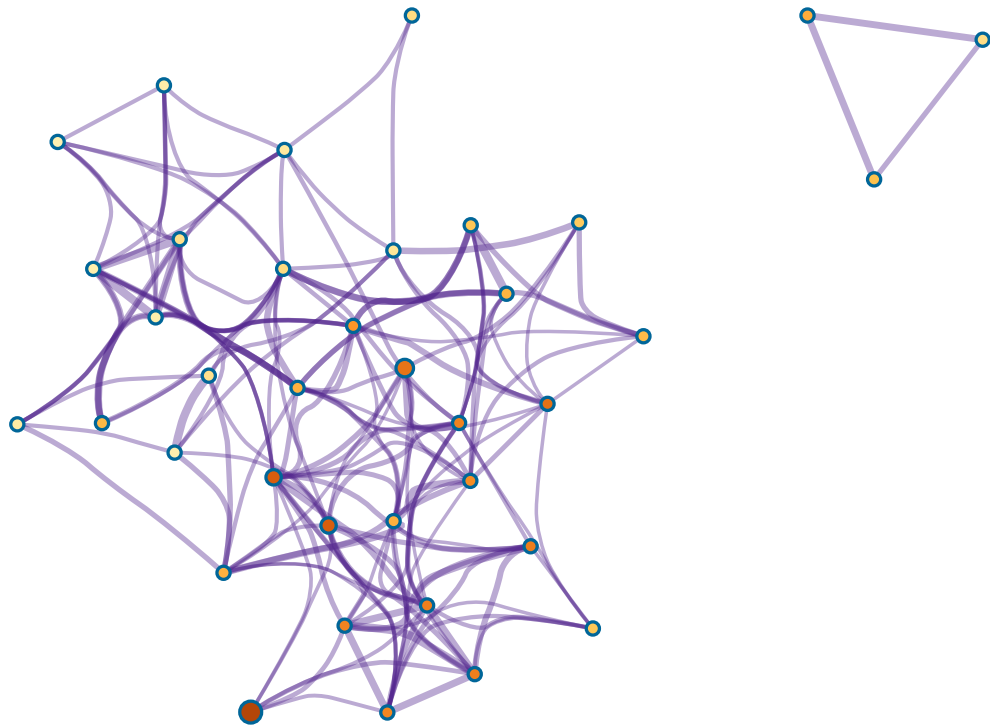

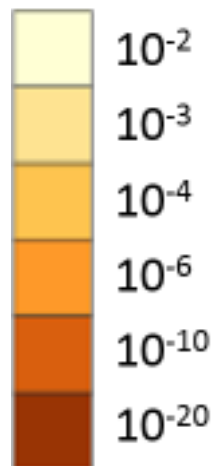

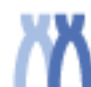 created by  
<http://metascape.org>

Supplement: S1 File — All raw data required to replicate the results of study were listed in this file. (ZIP) [file pone.0305343.s002.zip › original data/KEGG/Enrichment_GO/ColorByPValue.pdf]

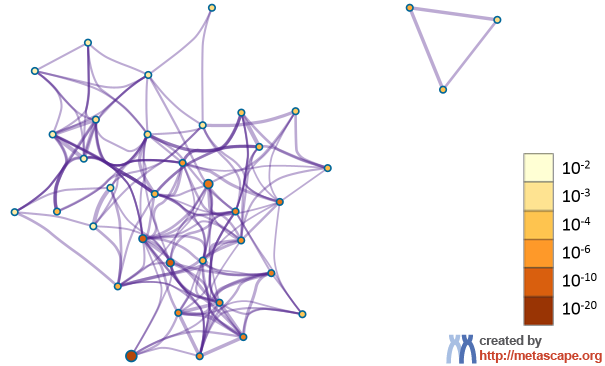

Supplement: S1 File — All raw data required to replicate the results of study were listed in this file. (ZIP) [file pone.0305343.s002.zip › original data/KEGG/Enrichment_GO/ColorByPValue.png]

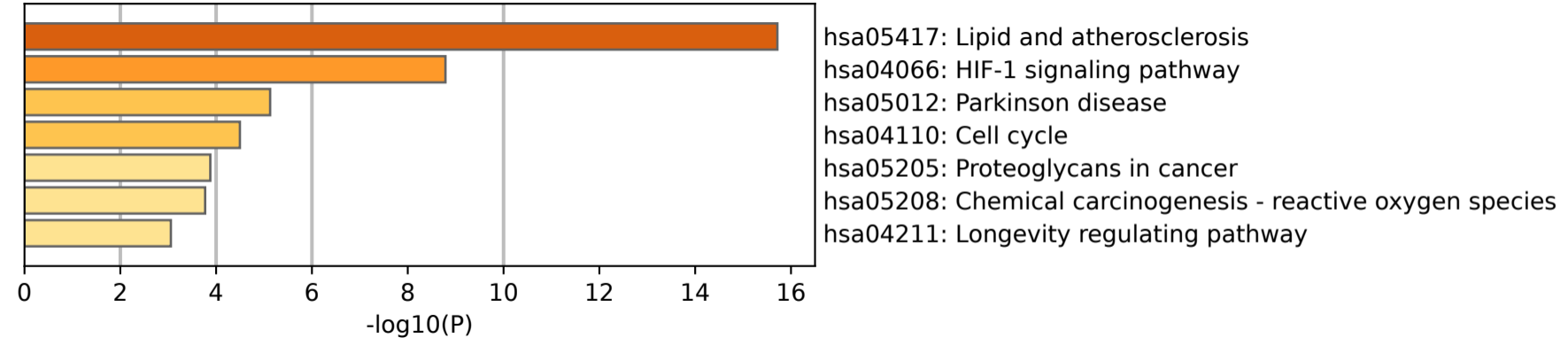

Supplement: S1 File — All raw data required to replicate the results of study were listed in this file. (ZIP) [file pone.0305343.s002.zip › original data/KEGG/Enrichment_heatmap/HeatmapSelectedGO.pdf]

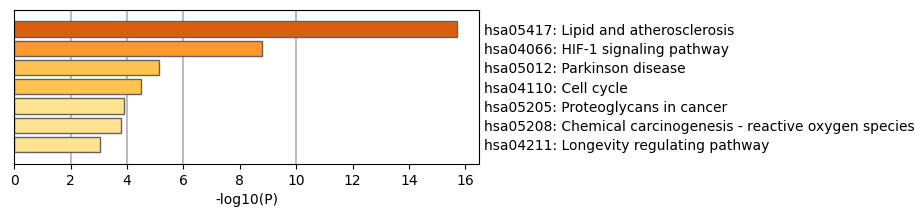

Supplement: S1 File — All raw data required to replicate the results of study were listed in this file. (ZIP) [file pone.0305343.s002.zip › original data/KEGG/Enrichment_heatmap/HeatmapSelectedGO.png]

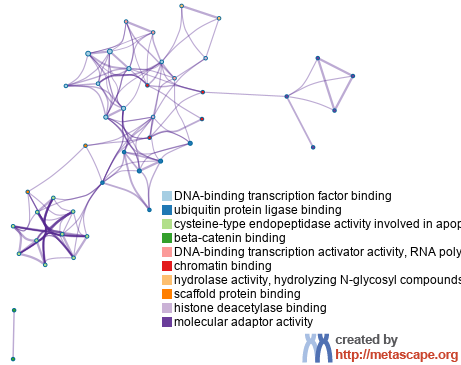

Supplement: S1 File — All raw data required to replicate the results of study were listed in this file. (ZIP) [file pone.0305343.s002.zip › original data/MF/Enrichment_GO/ColorByCluster.png]

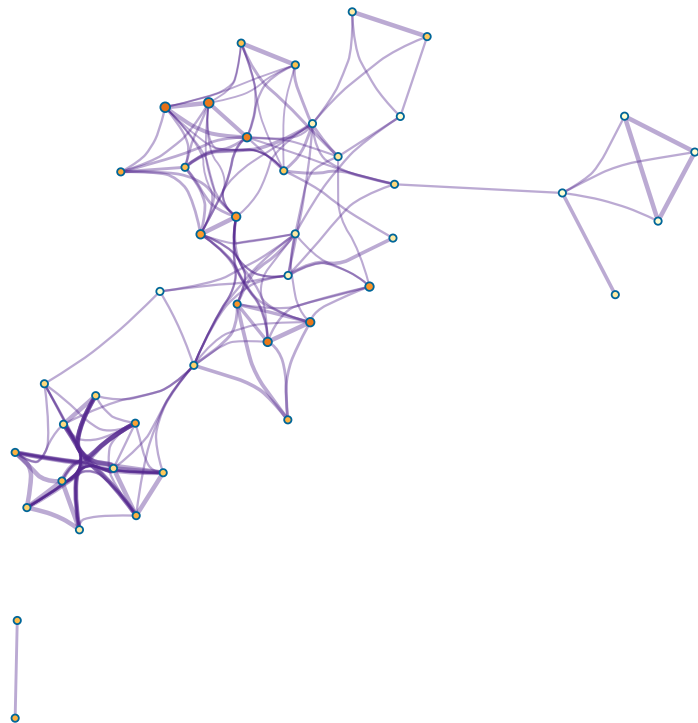

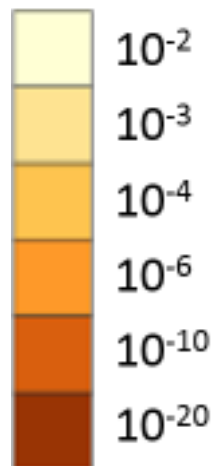

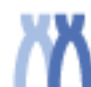 created by  
<http://metascape.org>

Supplement: S1 File — All raw data required to replicate the results of study were listed in this file. (ZIP) [file pone.0305343.s002.zip › original data/MF/Enrichment_GO/ColorByPValue.pdf]

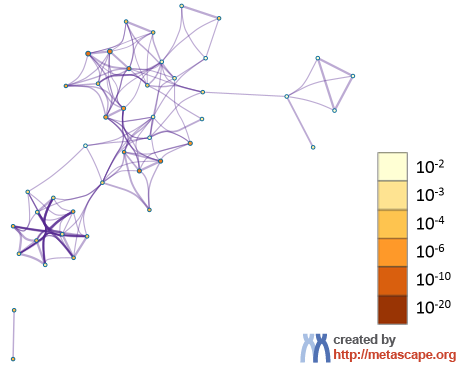

Supplement: S1 File — All raw data required to replicate the results of study were listed in this file. (ZIP) [file pone.0305343.s002.zip › original data/MF/Enrichment_GO/ColorByPValue.png]

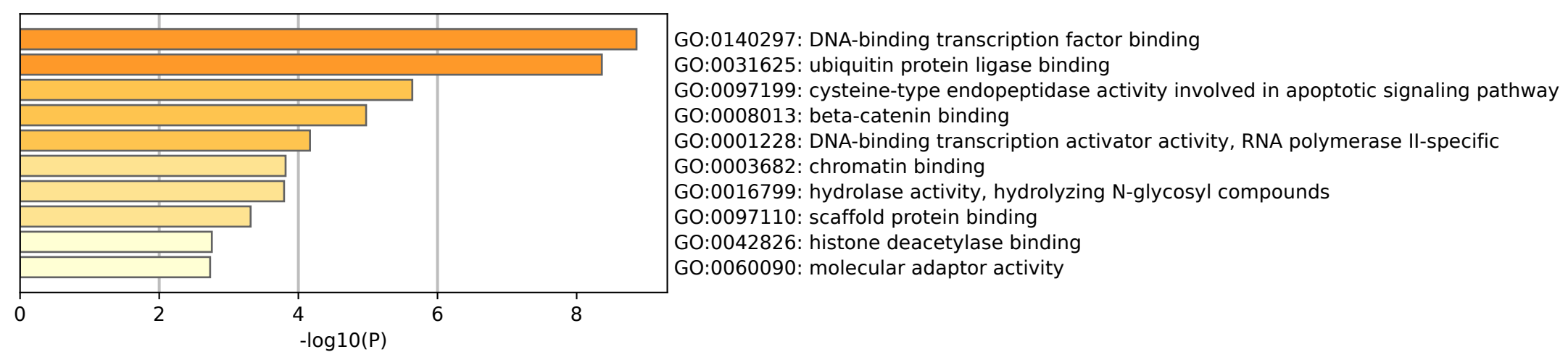

Supplement: S1 File — All raw data required to replicate the results of study were listed in this file. (ZIP) [file pone.0305343.s002.zip › original data/MF/Enrichment_heatmap/HeatmapSelectedGO.pdf]

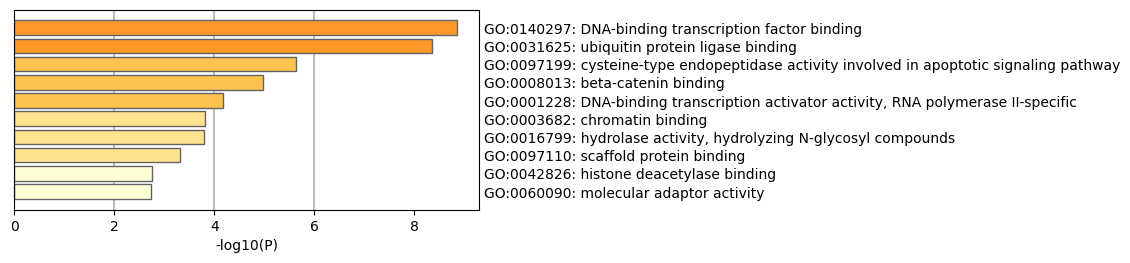

Supplement: S1 File — All raw data required to replicate the results of study were listed in this file. (ZIP) [file pone.0305343.s002.zip › original data/MF/Enrichment_heatmap/HeatmapSelectedGO.png]

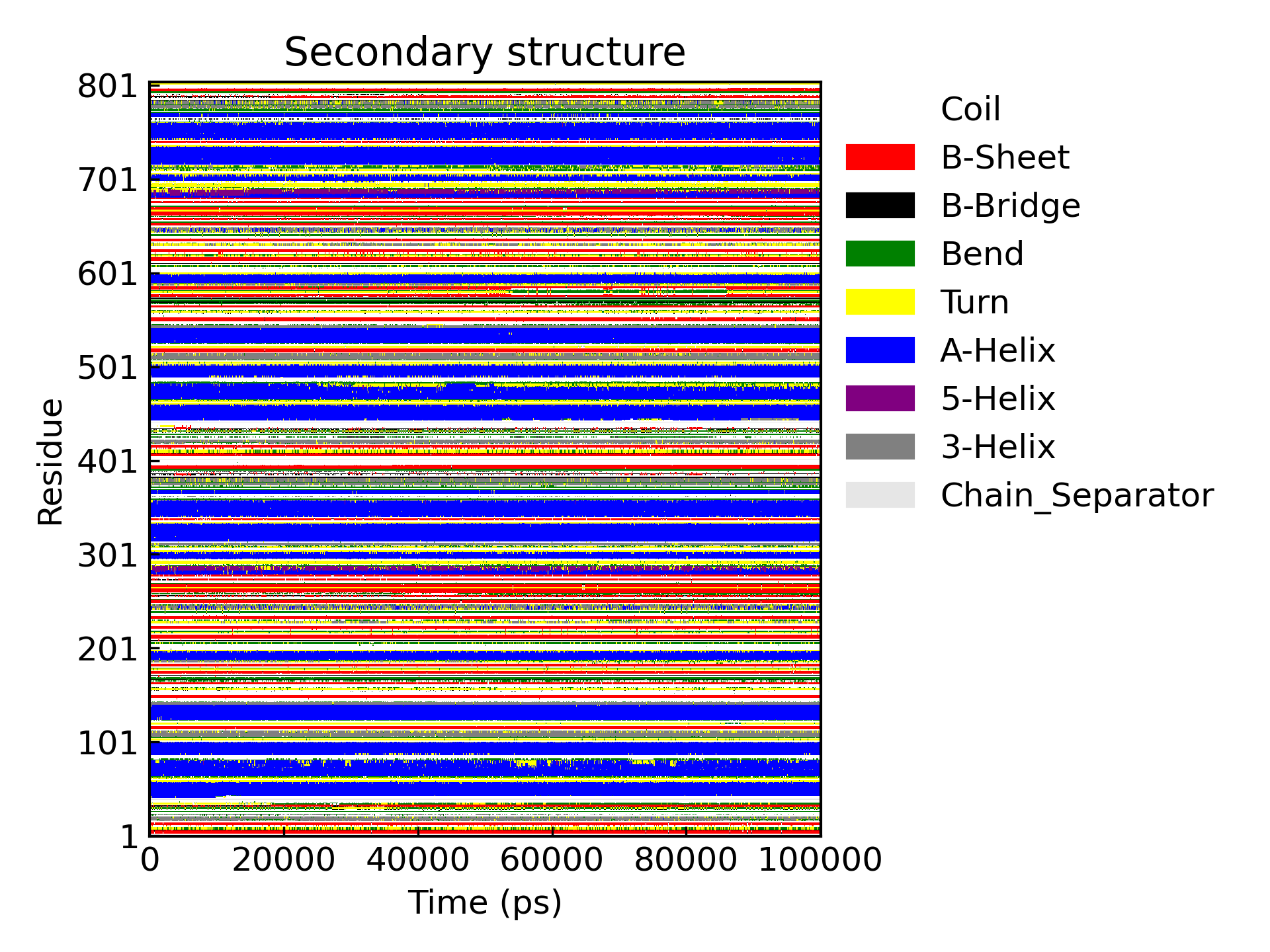

Supplement: S1 File — All raw data required to replicate the results of study were listed in this file. (ZIP) [file pone.0305343.s002.zip › original data/molecular dynamic/1m9k_NOS3_/secondary-structure.png]

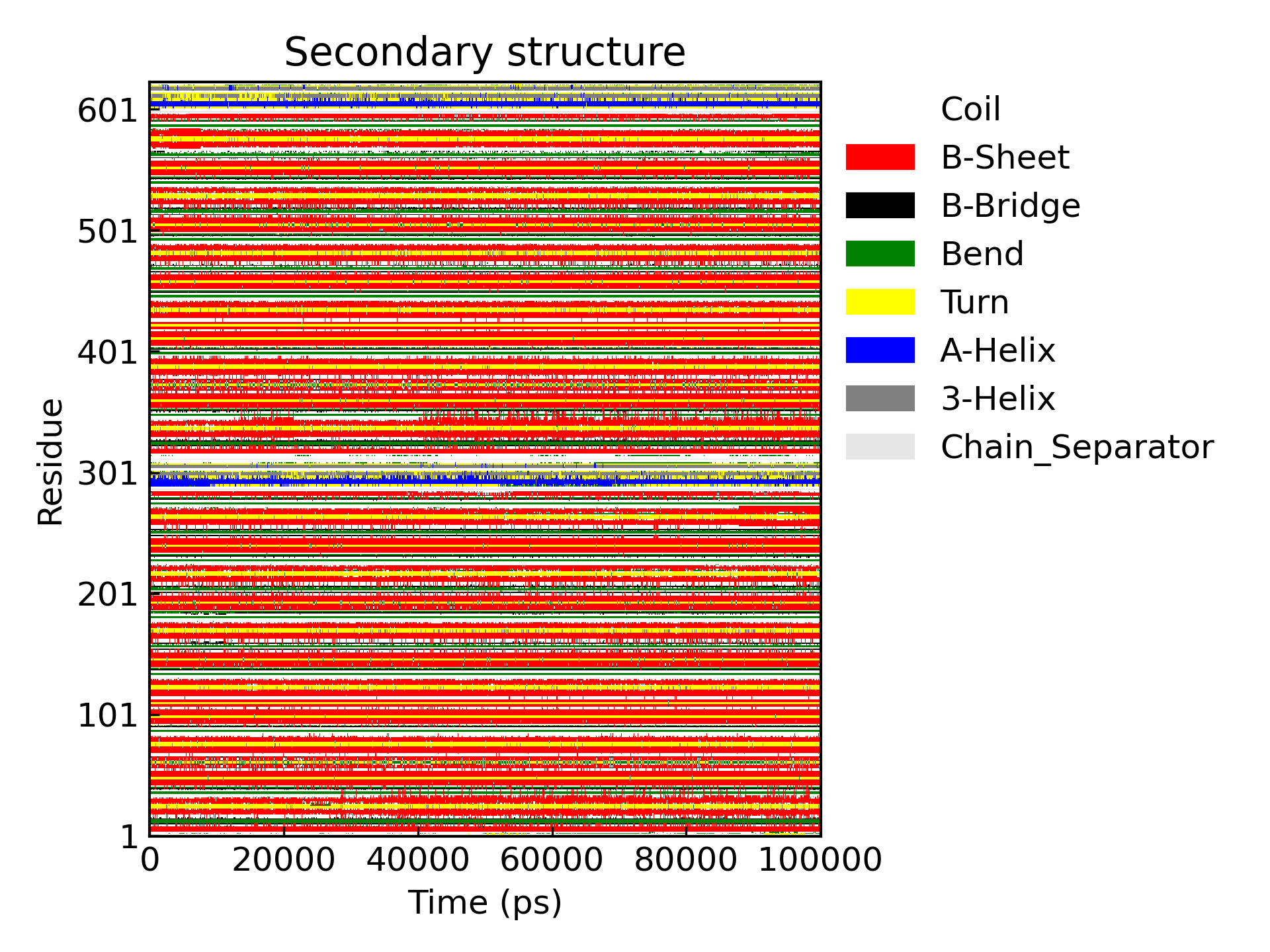

Supplement: S1 File — All raw data required to replicate the results of study were listed in this file. (ZIP) [file pone.0305343.s002.zip › original data/molecular dynamic/3wn7_keap1NRF2_/secondary-structure.png]

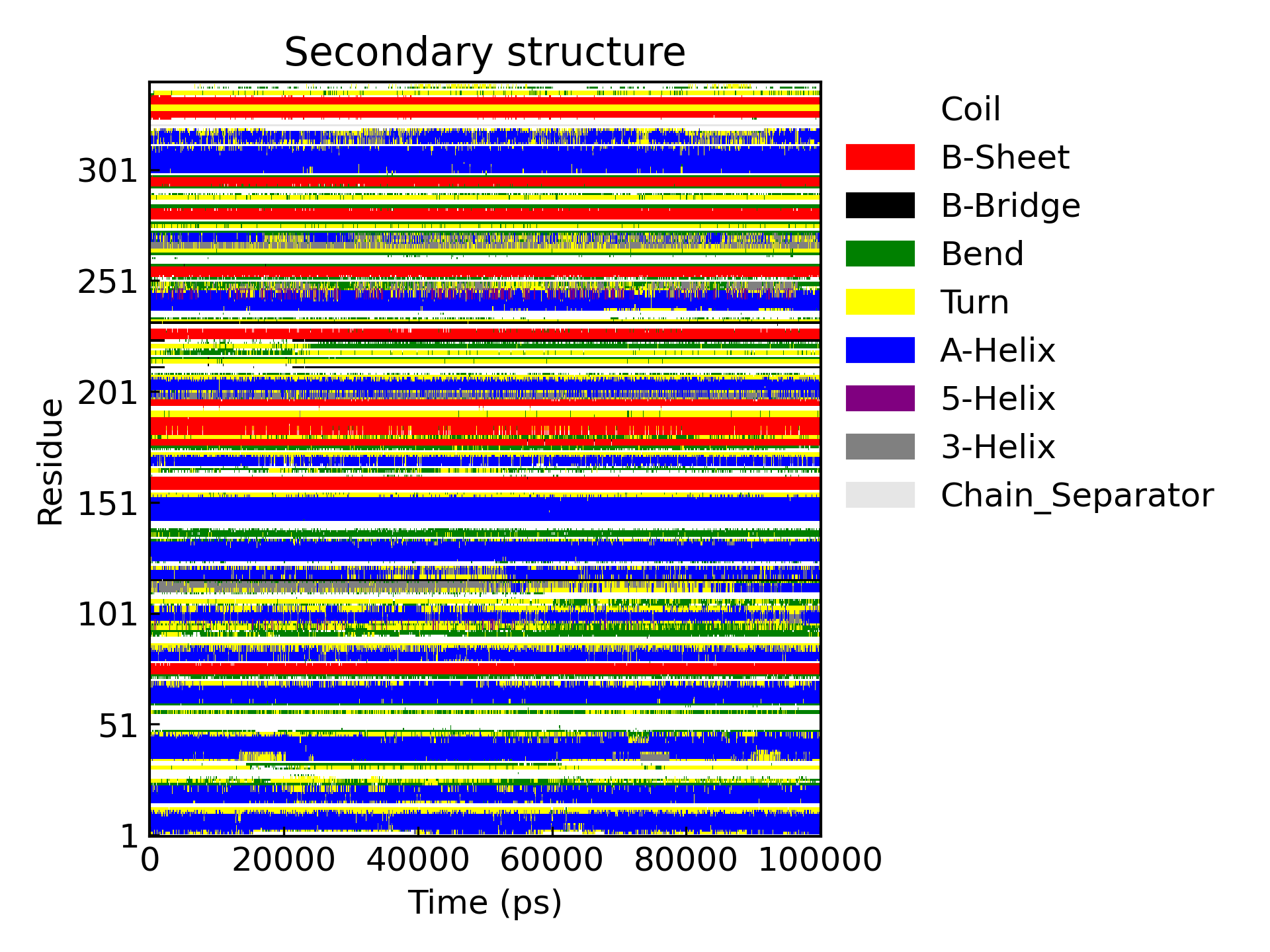

Supplement: S1 File — All raw data required to replicate the results of study were listed in this file. (ZIP) [file pone.0305343.s002.zip › original data/molecular dynamic/4zzh-SIRT1_/secondary-structure.png]
