# Supplementary material for: Network pharmacology and molecular-docking-based strategy to explore the potential mechanism of salidroside-inhibited oxidative stress in retinal ganglion cell
Source: PLoS One. 2024 Jul 5;19(7):e0305343. doi: 10.1371/journal.pone.0305343 (PMC11226129; doi:10.1371/journal.pone.0305343)
Supplement: S1 File — All raw data required to replicate the results of study were listed in this file. (ZIP) [file pone.0305343.s002.zip › original data/BP/Enrichment_GO/ColorByCluster.pdf]

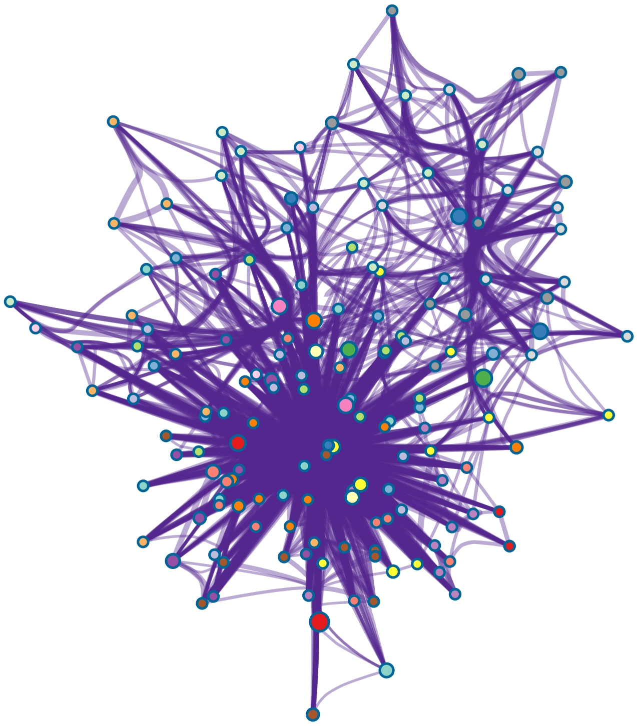

- response to inorganic substance
- cellular response to abiotic stimulus
- regulation of cellular response to stress
- cellular response to chemical stress
- negative regulation of intracellular signal transductior
- regulation of neuron death
- ovulation cycle
- negative regulation of catalytic activity
- cellular response to biotic stimulus
- heart development
- cellular response to organonitrogen compound
- regulation of response to cytokine stimulus
- protein catabolic process
- response to peptide
- positive regulation of transcription from RNA polymera
- negative regulation of transferase activity
- regulation of small molecule metabolic process
- regulation of cysteine-type endopeptidase activity inv
- positive regulation of protein catabolic process
- positive regulation of miRNA metabolic process

created by
http://metascape.org
