# Supplementary material for: Network pharmacology and molecular-docking-based strategy to explore the potential mechanism of salidroside-inhibited oxidative stress in retinal ganglion cell
Source: PLoS One. 2024 Jul 5;19(7):e0305343. doi: 10.1371/journal.pone.0305343 (PMC11226129; doi:10.1371/journal.pone.0305343)
Supplement: S1 File — All raw data required to replicate the results of study were listed in this file. (ZIP) [file pone.0305343.s002.zip › original data/CC/Enrichment_GO/ColorByCluster.pdf]

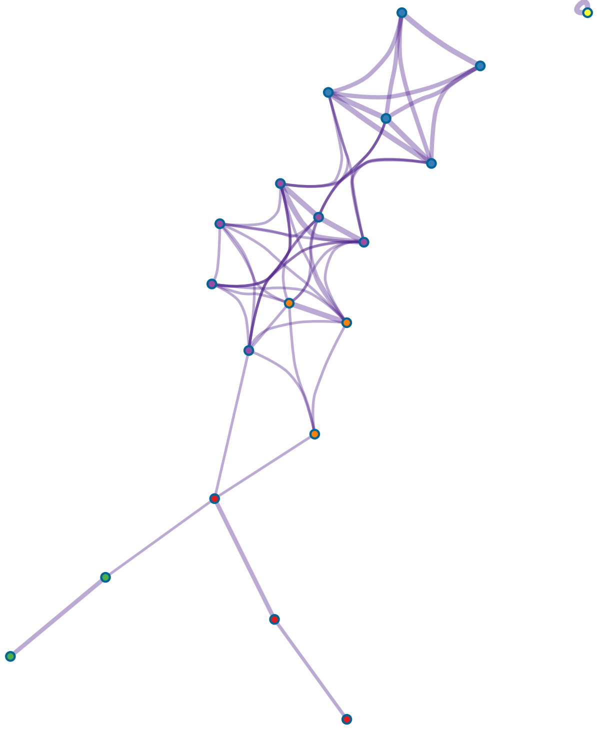

- euchromatin
- death-inducing signaling complex
- centrosome
- mitochondrial outer membrane
- presynapse
- Golgi membrane

created by
http://metascape.org
