# Supplementary material for: Network pharmacology and molecular-docking-based strategy to explore the potential mechanism of salidroside-inhibited oxidative stress in retinal ganglion cell
Source: PLoS One. 2024 Jul 5;19(7):e0305343. doi: 10.1371/journal.pone.0305343 (PMC11226129; doi:10.1371/journal.pone.0305343)
Supplement: S1 File — All raw data required to replicate the results of study were listed in this file. (ZIP) [file pone.0305343.s002.zip › original data/KEGG/AnalysisReport.pptx]

## Slide 1
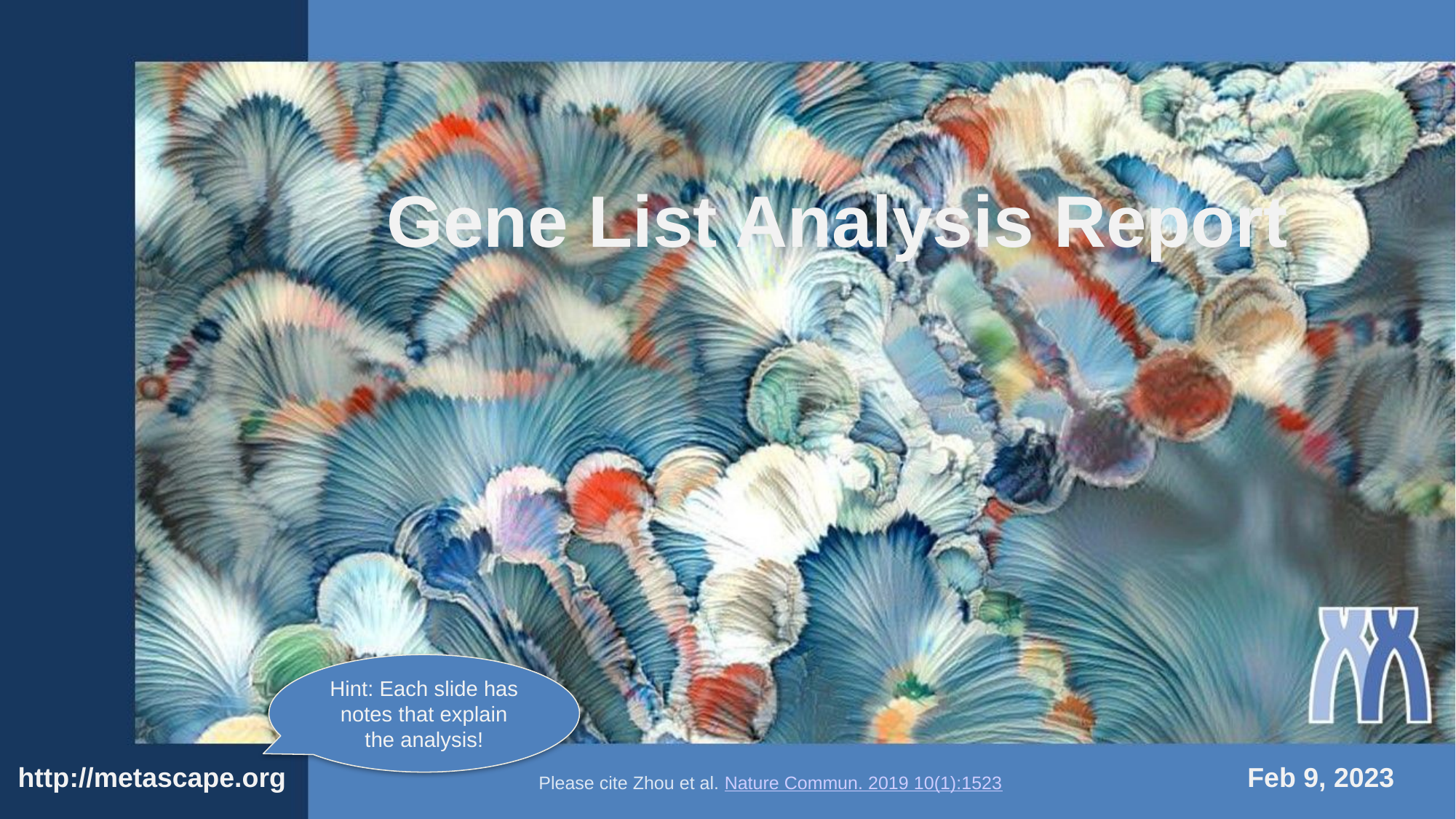

Gene List Analysis Report
Hint: Each slide has notes that explain the analysis!
http://metascape.org
Feb 9, 2023
Please cite Zhou et al. Nature Commun. 2019 10(1):1523

## Slide 2
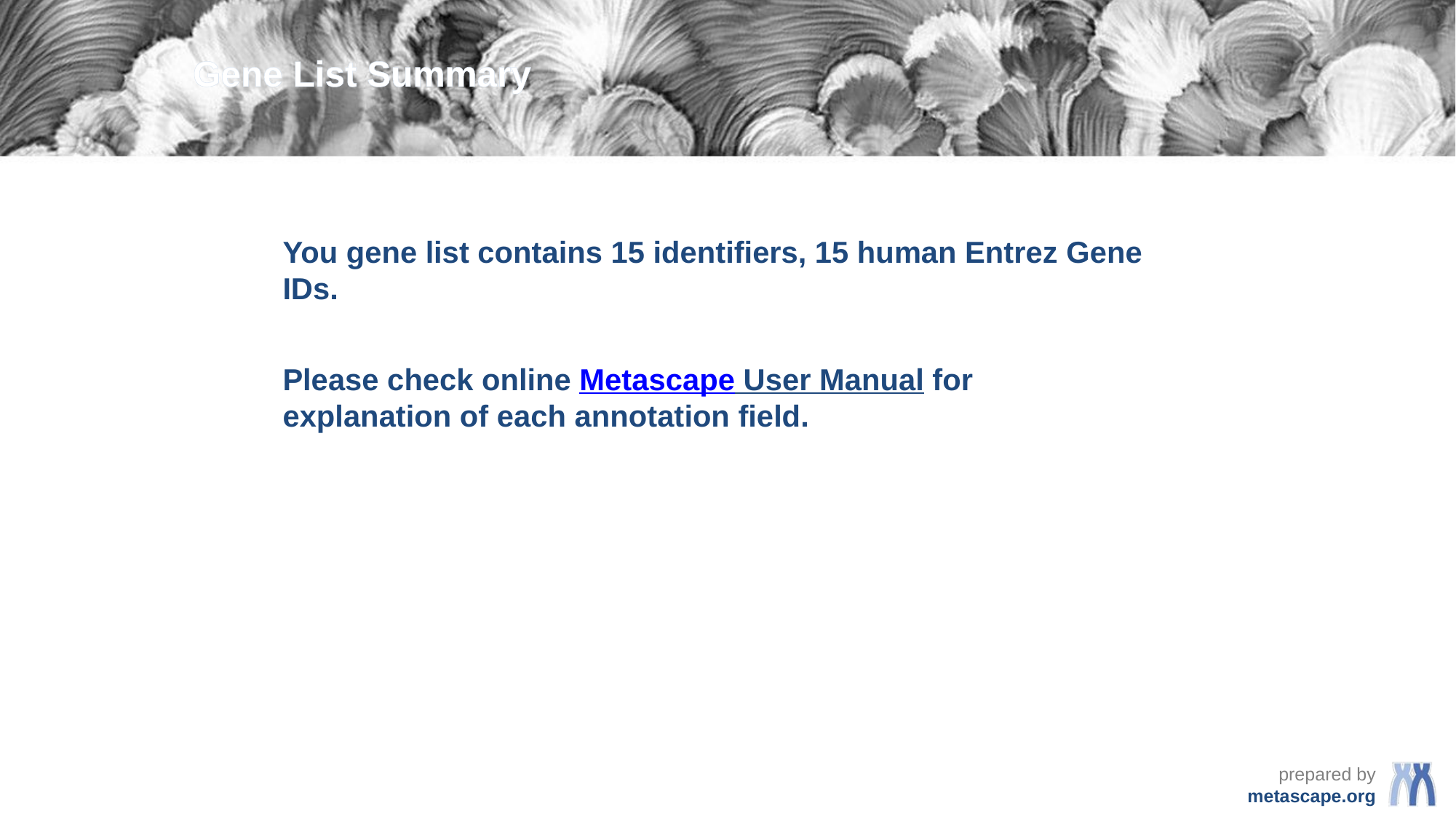

Gene List Summary
You gene list contains 15 identifiers, 15 human Entrez Gene IDs.
Please check online Metascape User Manual for explanation of each annotation field.

## Slide 3
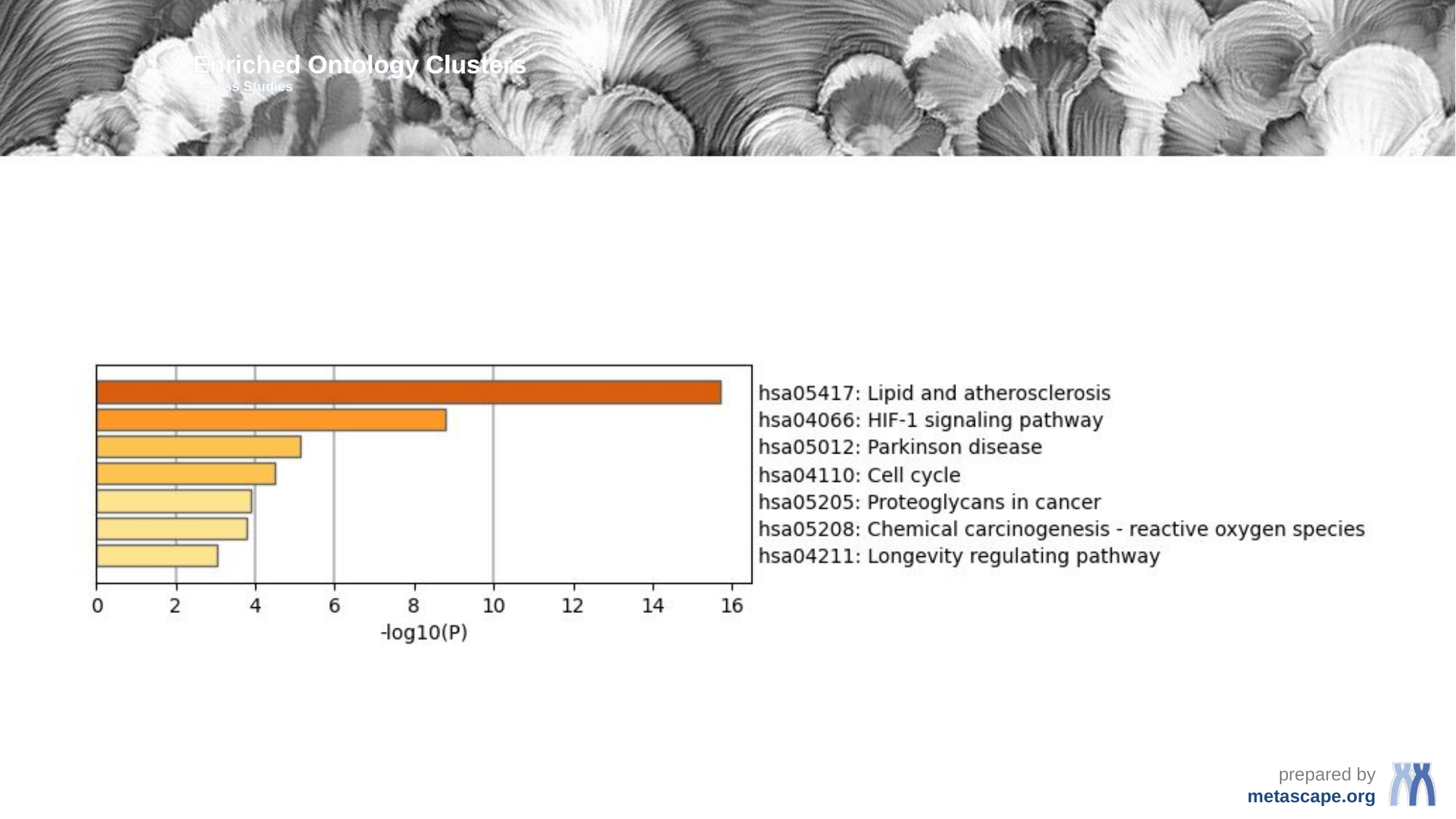

Enriched Ontology Clusters
Across Studies

## Slide 4
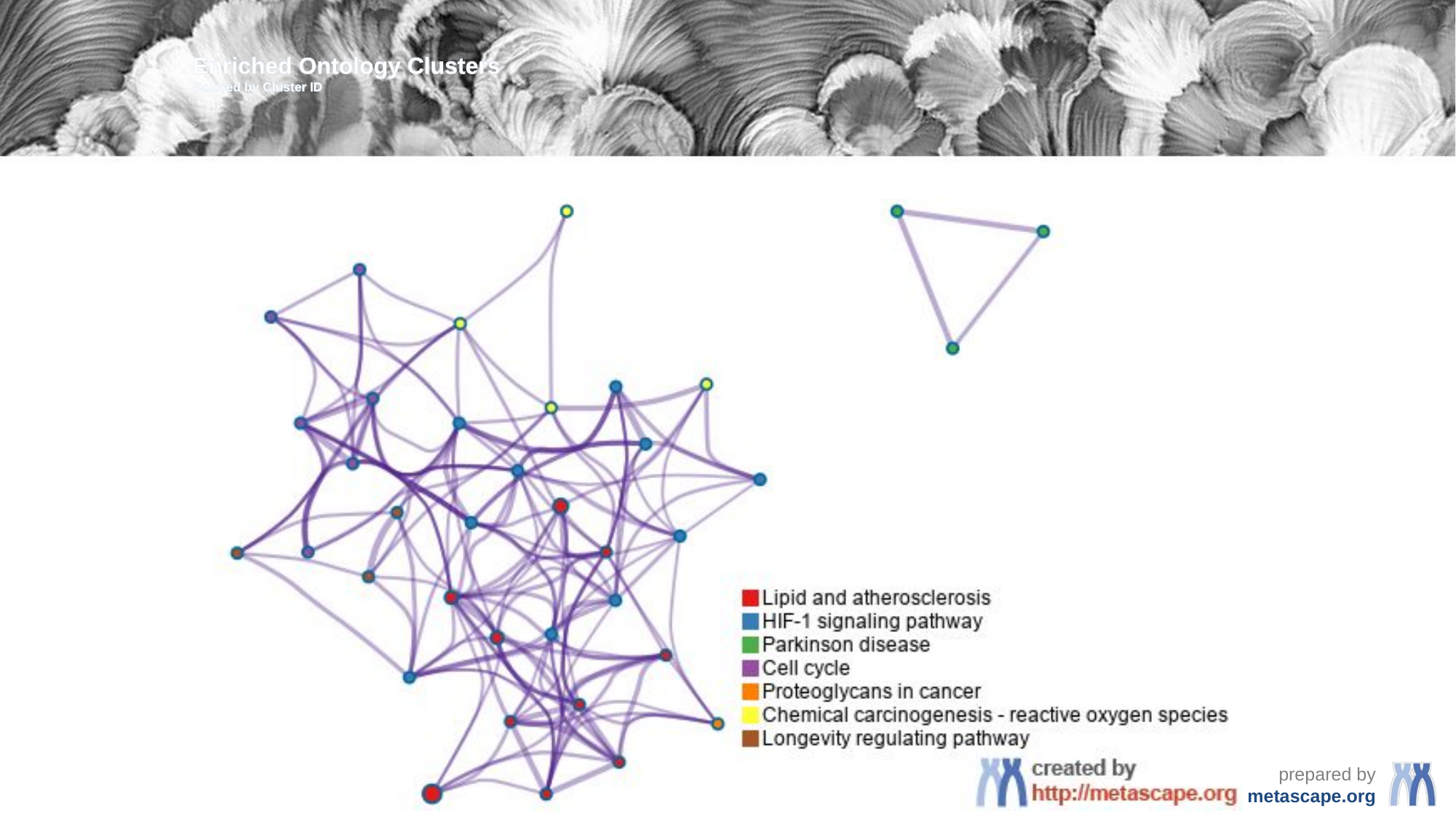

Enriched Ontology Clusters
Colored by Cluster ID

## Slide 5
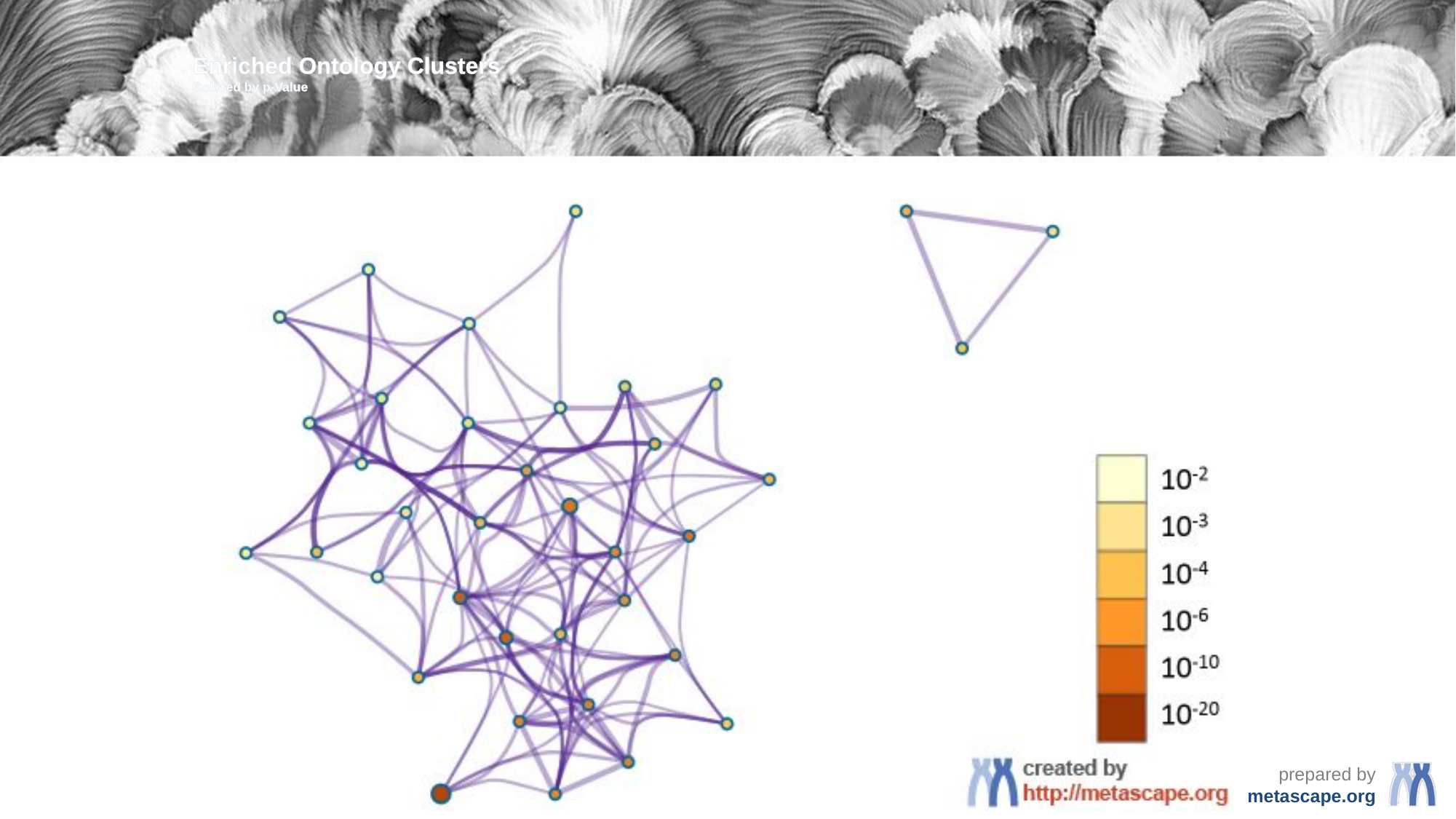

Enriched Ontology Clusters
Colored by p-Value

## Slide 6
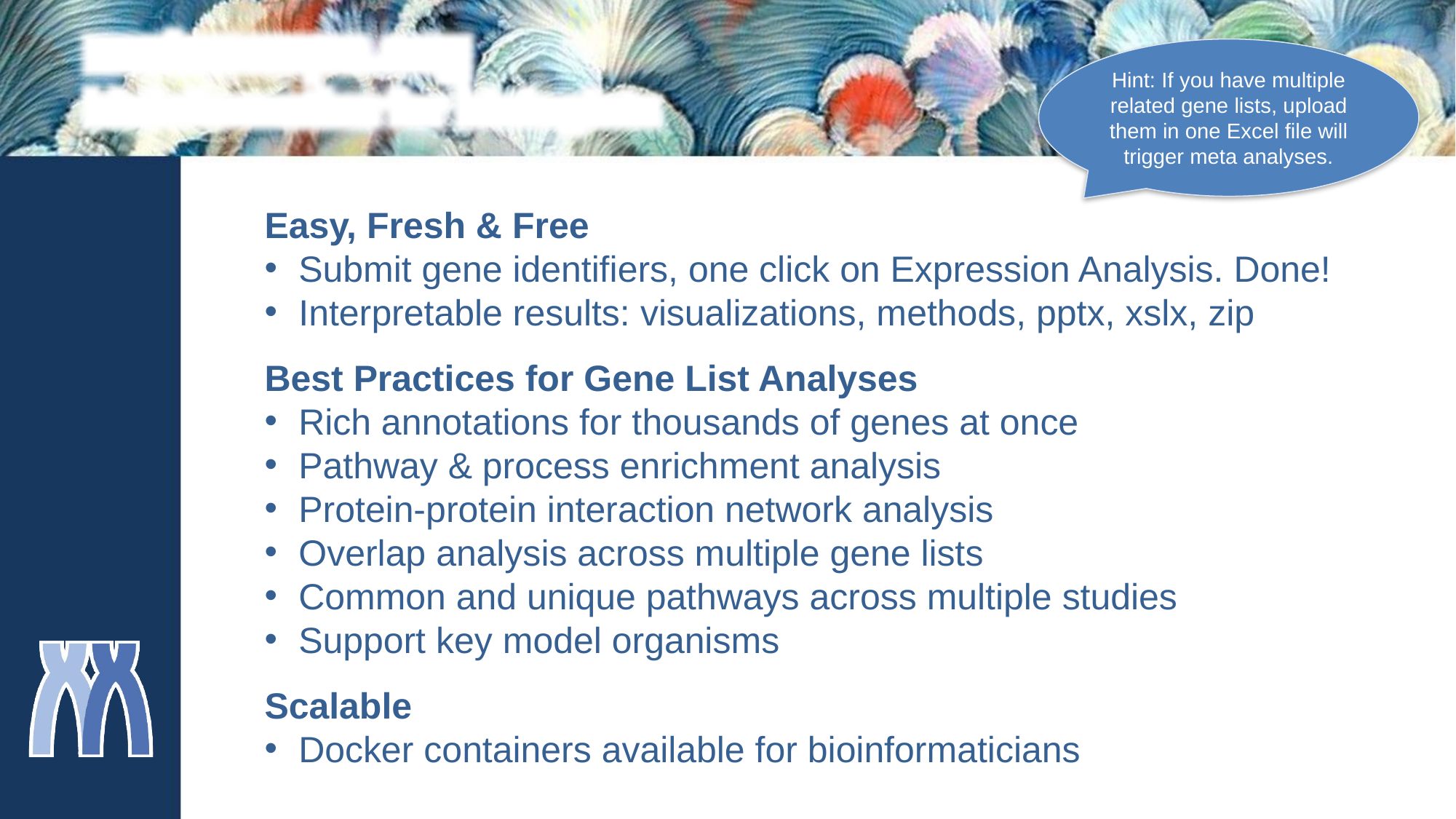

metascape.org
bioinformatics for biologists
Hint: If you have multiple related gene lists, upload them in one Excel file will trigger meta analyses.
Easy, Fresh & Free
Submit gene identifiers, one click on Expression Analysis. Done!
Interpretable results: visualizations, methods, pptx, xslx, zip
Best Practices for Gene List Analyses
Rich annotations for thousands of genes at once
Pathway & process enrichment analysis
Protein-protein interaction network analysis
Overlap analysis across multiple gene lists
Common and unique pathways across multiple studies
Support key model organisms
Scalable
Docker containers available for bioinformaticians
