# Supplementary material for: Network pharmacology and molecular-docking-based strategy to explore the potential mechanism of salidroside-inhibited oxidative stress in retinal ganglion cell
Source: PLoS One. 2024 Jul 5;19(7):e0305343. doi: 10.1371/journal.pone.0305343 (PMC11226129; doi:10.1371/journal.pone.0305343)
Supplement: S1 File — All raw data required to replicate the results of study were listed in this file. (ZIP) [file pone.0305343.s002.zip › original data/MF/Enrichment_GO/ColorByCluster.pdf]

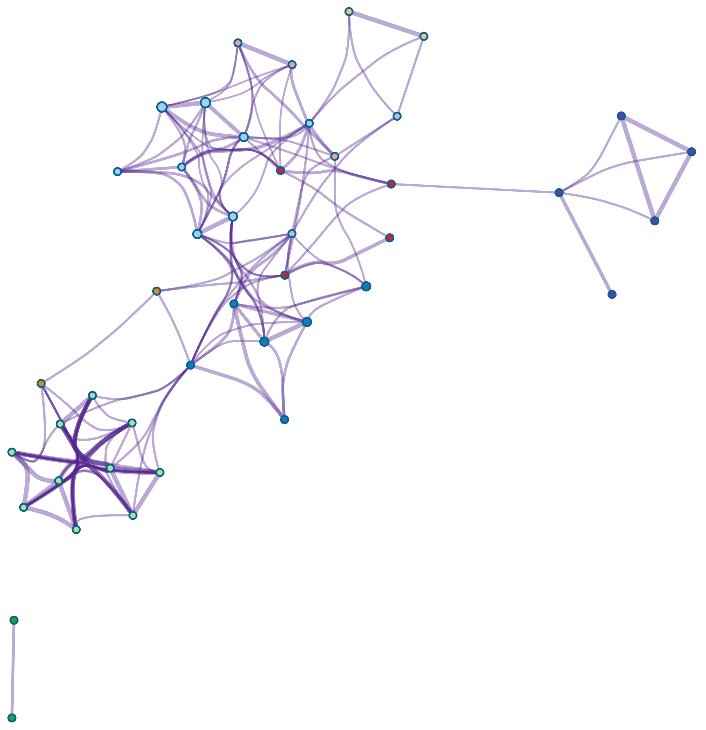

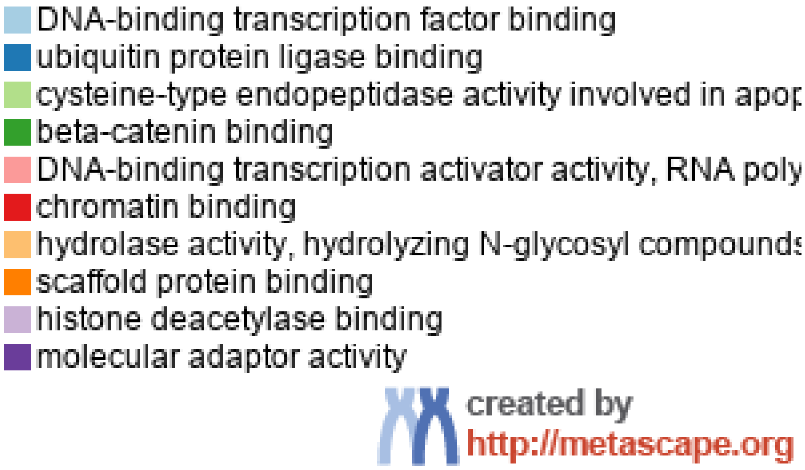
DNA-binding transcription factor binding
ubiquitin protein ligase binding
cysteine-type endopeptidase activity involved in apop
beta-catenin binding
DNA-binding transcription activator activity, RNA poly
chromatin binding
hydrolase activity, hydrolyzing N-glycosyl compounds
scaffold protein binding
histone deacetylase binding
molecular adaptor activity
created by
http://metascape.org
