# Supplementary material for: Network pharmacology and molecular-docking-based strategy to explore the potential mechanism of salidroside-inhibited oxidative stress in retinal ganglion cell
Source: PLoS One. 2024 Jul 5;19(7):e0305343. doi: 10.1371/journal.pone.0305343 (PMC11226129; doi:10.1371/journal.pone.0305343)
Supplement: Attachment — Submitted filename: Response to Reviewers.docx [file pone.0305343.s003.docx]

Dear Editors and Reviewers:

Thank you for your letter and for the reviewers’ comments concerning our manuscript

entitled “Network pharmacology and molecular-docking-based strategy to explore the potential mechanism of salidroside-inhibited oxidative stress in retinal ganglion cell” (PONE-D-24-07716). Those comments are all valuable and very helpful for revising and improving our paper, as well as the important guiding significance to our researches. We have studied comments carefully and have made correction which we hope meet with approval. The reviewer comments are laid out below in *italicized* *font* and specific concern shave been numbered. Our response is given in normal font and changes/additions to the manuscript are given in red text.

Responds to the reviewer’s comments:

*1. Please ensure that your manuscript meets PLOS ONE's style requirements, including those for file naming. The PLOS ONE style templates can be found at*

*https://journals.plos.org/plosone/s/file?id=wjVg/PLOSOne_formatting_sample_main_body.pdf and*

*https://journals.plos.org/plosone/s/file?id=ba62/PLOSOne_formatting_sample_title_authors_affiliations.pdf*

Author response: Thank you for your review. We have modified the citation style and other content as required.

*2. We note that the grant information you provided in the ‘Funding Information’ and ‘Financial Disclosure’ sections do not match.*

*When you resubmit, please ensure that you provide the correct grant numbers for the awards you received for your study in the ‘Funding Information’ section.*

Author response: Thank you for your review. We made sure to provide the research grant number received in the Funding Information section.

*3. We note that your Data Availability Statement is currently as follows: [All relevant data are within the manuscript and its Supporting Information files.]*

*Please confirm at this time whether or not your submission contains all raw data required to replicate the results of your study. Authors must share the “minimal data set” for their submission. PLOS defines the minimal data set to consist of the data required to replicate all study findings reported in the article, as well as related metadata and methods (https://journals.plos.org/plosone/s/data-availability#loc-minimal-data-set-definition).*

Author response: We apologize for any omissions in the uploaded content. We have uploaded the original data that can reproduce the research results as supporting information files. If there are any missing data, please let us know in time.

4. *Please ensure that you include a title page within your main document. You should list all authors and all affiliations as per our author instructions and clearly indicate the corresponding author.*

Author response: Thank you for your feedback. We have added the title page to the main document as requested.

5. *We note that Figure 1 in your submission contain copyrighted images. All PLOS content is published under the Creative Commons Attribution License (CC BY 4.0), which means that the manuscript, images, and Supporting Information files will be freely available online, and any third party is permitted to access, download, copy, distribute, and use these materials in any way, even commercially, with proper attribution. For more information, see our copyright guidelines: http://journals.plos.org/plosone/s/licenses-and-copyright.*

Author response: Sorry for the oversight here, we have modified Figure 1 without changing the original meaning.

*Additional Editor Comments:*

*There is a validation step required with MD simulations > 100 nano seconds and WT-MetaD simulations for >50 nano seconds for the docking results.*

Author response: Thank you for your review. To solve this problem, three new authors joined our team. MOE2019 was used for molecular docking experiments. After docking the protein and small molecule, the optimal complex conformation was selected according to the binding score, and Gromacs2018 software was used for 100ns dynamic simulation. This part is supplemented in the methods and results section.

Reviewer #1:

1. *I believe that the manuscript is in general sound and does come to reasonable conclusions. However, the molecular docking portion of the paper seems to be incomplete. Docking alone, via Autodock, does not provide enough adequate support for the conclusions in this manuscript. Follow-up with molecular dynamic (MD) will provide more information to support the authors claims, via free energy calculations, e.g. MM/PBSA. I have listed some publications for the authors review.*

Author response: Thank you for your review. To solve this problem, three new authors joined our team. MOE2019 was used for molecular docking experiments. After docking the protein and small molecule, the optimal complex conformation was selected according to the binding score, and Gromacs2018 software was used for 100ns dynamic simulation. This part is supplemented in the methods and results section.

Reviewer #2:

1. *Please check the formatting in manuscript thoroughly and add the spaces wherever needed.*

Author response: Thanks for your review, we added spaces on line 21 and elsewhere where necessary.

2. *Fig. 2c and 3d; mention X and Y axis descriptions in detail.*

Author response: Thank you for your suggestion, we have added the description at lines 258 and 259.

3. *The format of references is different from the manuscript body. Please check it.*

Author response: We have revised the format of the references to be consistent with the main text.

4. *Use subscript in the chemical formula.*

Author response: We modified the chemical formula in line 107.

5. *In Line 224 and 228; check the spelling of SAL.*

Author response: Thank you for your review, we have changed the spelling of SAL.

6. *In Material and method section; authors have referred “1.2.2” but there is no such explanation to it.*

Author response: Sorry for the writing error here, we have changed 1.2.2 to 2.2 and marked it in red font.

7. *Elaborate the abbreviations used in the manuscript.*

Author response: We have added the full name of the abbreviation in lines 183, 187, 188, 195, 196 and marked them in red font.

8. *Some grammatical errors in Line 290 and 304.*

Author response: Thank you for your review. We have modified 3.4 GO and KEGG Enrichment Analysis and marked it in red font.

**Special thanks to you for your good comments.**
