# Supplementary material for: Network pharmacology and molecular-docking-based strategy to explore the potential mechanism of salidroside-inhibited oxidative stress in retinal ganglion cell
Source: PLoS One. 2024 Jul 5;19(7):e0305343. doi: 10.1371/journal.pone.0305343 (PMC11226129; doi:10.1371/journal.pone.0305343)
Supplement: Attachment — Submitted filename: Response to Reviewers.docx [file pone.0305343.s004.docx]

Response to Reviewers

Dear Editors:

Thank you for your letter and for the comments concerning our manuscript entitled “Network pharmacology and molecular-docking-based strategy to explore the potential mechanism of salidroside-inhibited oxidative stress in retinal ganglion cell”(PONE-D-24-07716R1). We have studied comments carefully and have made correction which we hope meet with approval. The reviewer comments are laid out below in italicized font and specific concern shave been numbered. Our response is given in normal font and changes/additions to the manuscript are given in red text. **We have made changes to our financial disclosures and included an updated statement in the cover letter.**

*Additional Editor Comments:*

*Authors have done commendable Job revising the manuscript. Most of the comments have been addressed. RMSD of MD simulations does not reveal the binding stress for neighboring amino acid side chain residues. It is important to calculate the strain on the components of the receptor pocket to reach a conclusion regarding validity of the proposed protein ligand interaction. Even if authors cannot do WT-MetaD simulations they should at least perform mmPBSA calculations with their trajectories. It's quite simple to perform and is an adjunct analysis on already done simulations (Ref. 10.1021/acs.jctc.1c00645 & 10.1016/j.cpc.2014.09.010).*

*Free energy calculations validate the MD simulations and are a necessary step and are not difficult to perform.*

Author response： Dear Editor, thank you for your suggestions. We have added the description of MMPBSA calculations in the Methods (2.12) section and made changes in the Results (3.6-3.7). The changes are marked in red font.

**Special thanks to you for your good comments.**
